# Identification of the need for home visiting nurse: development of a new assessment tool

**DOI:** 10.5334/ijic.1159

**Published:** 2014-03-13

**Authors:** Atsuko Taguchi, Satoko Nagata, Takashi Naruse, Yuki Kuwahara, Takuhiro Yamaguchi, Sachiyo Murashima

**Affiliations:** Division of Community Health Care System, Health Sciences, Tohoku University Graduate School of Medicine, Sendai, Japan; Department of Community Health nursing, Graduate School of Medicine, The University of Tokyo, Tokyo, Japan; Department of Community Health nursing, Graduate School of Medicine, The University of Tokyo, Tokyo, Japan; Department of Community Health Nursing, Graduate School of Medicine, The University of Tokyo, Tokyo, Japan; Division of Biostatistics, Tohoku University Graduate School of Medicine, Sendai, Japan; Oita University of Nursing and Health Science, President, 2944-9, Megusuno, Oita City, Oita, Japan

**Keywords:** home visiting nursing service, need assessment, assessment tool

## Abstract

**Objective:**

To develop a Home Visiting Nursing Service Need Assessment Form (HVNS-NAF) to standardize the decision about the need for home visiting nursing service.

**Methods:**

The sample consisted of older adults who had received coordinated services by care managers. We defined the need for home visiting nursing service by elderly individuals as the decision of the need by a care manager so that the elderly can continue to live independently. Explanatory variables included demographic factors, medical procedure, severity of illness, and caregiver variables. Multiple logistic regression was carried out after univariate analyses to decide the variables to include and the weight of each variable in the HVNS-NAF. We then calculated the sensitivity and specificity of each cutoff value, and defined the score with the highest sensitivity and specificity as the cutoff value.

**Results:**

Nineteen items were included in the final HVNS-NAF. When the cutoff value was 2 points, the sensitivity was 77.0%, specificity 68.5%, and positive predictive value 56.8%.

**Conclusions:**

HVNS-NAF is the first validated standard based on characteristics of elderly clients who required home visiting nursing service. Using the HVNS-NAF may result in reducing the unmet need for home visiting nursing service and preventing hospitalization.

## Introduction

The increasing health-care cost for disabled elderly in developed countries is an important issue. Prolonging home care is an effective means of reducing health care costs [1, 2], as it allows elderly disabled persons to maintain their independence and, in many cases, their homes.

In Japan, where the elderly population is rising faster than in any other country [3], the public Long-Term Care Insurance system was established to support independence among the older population, in part by introducing home care services [4]. To ensure their independence and provide effective home care, Long-Term Care Insurance clients can tailor the services to conform to their own care plan. Usually, the care plan is made by care managers, who are professionals in the integrated care management of elderly individuals. The integrated care management process is comprised of intake, assessment, analysis of needs, care planning, and implementation of services, monitoring, and evaluation [5].

Among the Long-Term Care Insurance services, home visiting nursing service is one of the important services that maintain clients’ independence and continuous home care [6]. Since home visiting nursing service tends to be needed more among elderly who require medical treatment and assessment in home care [7], there has been a rising need for home visiting nursing service in Japan due to the aging population and the move toward deinstitutionalization. However, the use of home visiting nursing service in the Long-Term Care Insurance in 2010 increased by only 130% since 2001, whereas the use of other services increased by 170% during the same period [8, 9]. The fact that the use of home visiting nursing service has not risen as quickly as the use of other services could be due to one of two reasons: the demand for home visiting nursing service was already met and home visiting nursing service was not needed as much as other services, or the demand for home visiting nursing service was unmet and more home visiting nursing service resources and visits were needed. Home visiting nursing service provides proactive management of symptoms of older persons with disability. The older persons with disability would be able to continue to live at home, reducing the risk of readmission and lowering the cost of medical care. Reducing the unmet need for nurse home visiting can be expected to curb the growth of medical spending.

An unmet need for home visiting nursing service indicates a serious problem that must be addressed, whether or not any home visiting nursing service is currently being provided [10, 11]. If the need remains unmet, the elderly population may be at risk for a variety of adverse outcomes [12, 13]. For clients to obtain a positive outcome, nurses need sufficient time to provide direct care [14, 15]. Nagata et al. showed that among the Japanese frail elderly population, whose care need levels in the Long-Term Care Insurance system are classified as higher than level one, about 40% of those who needed home visiting nursing service did not use it [16]. Nagata et al. stressed the importance of developing strategies for encouraging the use of home visiting nursing service by nonusers. To reduce the unmet needs for home visiting nursing service, it is important to reach an appropriate decision with regard to the need for home visiting nursing service in care planning. It is particularly difficult for care managers who are novices and have little knowledge of home visiting nursing service, to make an appropriate decision about whether particular clients need home visiting nursing service.

Many tools of comprehensive assessment of older people have been developed in the past. Assessment tools that are widely used are the Omaha System [17], Minimum Data Set-Home Care (MDS-HC) [18], Outcome and Assessment Information Set (OASIS) [19], and Camberwell Assessment of Need for Elderly (CANE) [20]. These assessment tools can comprehensively assess the physical and mental condition and the living conditions of elderly people living at home, and clarify their health problems and needs. These assessment tools cannot clarify the types of services needed to solve their health problems. The precise choice of services influences the outcome of the health of elderly people [13]. Since there is an unmet need of home visiting nursing service, it is especially significant to standardize the decision about the need for home visiting nursing service [21, 22]. Standardization of the decision about the need for home visiting service would lead to further improvement of integrated care for older people.

The aims of this study were to clarify the characteristics of clients who need home visiting nursing service and to develop a Home Visiting Nursing Service Need Assessment Form (HVNS-NAF) in an effort to standardize the decision about the need for home visiting nursing service.

## Japanese long-term care insurance system

The term “insured persons” in the long-term care insurance system refers to all individuals aged 40 years and above nationwide who have this insurance. Those aged 65 years and over are referred to as No. 1 insured persons and those aged 40–64 years who subscribe to medical care insurance as No. 2 insured persons. In the No. 1 group, recipients of long-term care services are all those in need of long-term care (those who have already reached such a condition) and all those in need of support (those who are in a condition where it is feared that they will require long-term care services in the future). In the No. 2 group, eligibility for benefits is restricted to those for whom long-term care is necessary due to an illness accompanying aging such as middle-aged dementia, stroke, and the like.

Services are provided in the form of benefits-in-kind (there are no cash benefits). For such services, the user is liable for 10% of the benefit amount. In addition, those admitted to facilities are liable for meal and daily living costs. Therefore, the financial burden for these users does not become intolerably high, and fixed amounts have been set for total patient cost-sharing. Should patient cost-sharing go beyond these limits, the amount in excess is to be compensated for as high-cost services for those in need of long-term care or for those in need of assistance at home.

In consultation with the care manager, a care plan is developed free of charge compatible with factors that include the extent of care required, the wishes of the elderly person concerned, and the family's situation. There is a limit to the amount that can be expended for home long-term care, so the services used must be within this range. The user is liable for 10% of the cost to be paid during the period the services are supplied. The home visiting nurse agency is one of the services available that provides long-term care. Home visiting nurses provide care for patients with chronic illness, patients with disability, and terminal patients who need palliative care.

## Methods

### Data collection

Data collection took place in 2009. The participants were care managers who coordinate comprehensive services for older people in Japan including the decision about the use of a home visiting nurse. Participants were recruited from the Welfare And Medical Service Network System (WAM NET) database in Japan.

For this study, eight districts in the following eight prefectures were selected: Miyagi, Chiba, Nagano, Shizuoka, Shiga, Shimane, Kagawa, and Fukuoka. These areas were selected based on the following methodology: (1) we ranked all 47 Japanese prefectures according to their utilization rate of home visiting nursing service for persons over 65 years old; (2) we created eight groups of prefectures, ranging from the prefecture group with very high utilization rate to the prefecture group with very low utilization rate; (3) we selected eight prefectures keeping in mind the feasibility of conducting a large sample survey; and (4) we selected the eight districts whose demographics were similar to those of the prefecture in which they were located.

The clients of all care manager agencies in the eight districts were included in this study. The clients had to be aged over 40 years and had to have received at least one visit from a home visiting nurse in November 2008. There were 553 care manager agencies in the eight prefectures. If the care managers at a care manager agency agreed to participate, the care managers gathered information on their clients, including demographic characteristics from charts and assessments of their service needs. Completed questionnaires were mailed back to the researcher. Each care manager was asked to sample one-fifth of their clients, i.e., they filled a questionnaire for every one out of five clients, from their clients that were listed in the order of the Japanese syllabary.

From the 256 care manager agencies (46.3%) that agreed to participate, data on 3606 clients were collected. Six hundred and sixty-nine clients were excluded due to incomplete data in which the participant did not answer the questions on need and utilization of home visiting nursing service, the type of qualification (medical or welfare) of the care manager, and years of experience of the care manager, leaving 2937 clients.

In this study, we defined a care manager who can assess the need for home visiting nursing service most reasonably as “a medical professional with 3 years or more experience.” Data on 1085 clients that were collected by care managers who fit this definition were utilized to create the assessment form.

This study was approved by the Ethics Committee of the Graduate School of Medicine, the University of Tokyo.

### Variables

Variables related to the need of home visiting nursing service were gathered from previous studies, textbooks, and written reports regarding home visiting nursing service. The variables were refined and grouped by similarity. At that time, we structured the variables especially around chronic illness because most long-term care patients have chronic conditions.

### Dependent variables

According to Bradshaw [23], a normative need is a need that is defined by professionals or experts according to their own standards. In this study, we adopted the definition of the need for home visiting nursing service as the need for home visiting nursing service by an elderly individual as determined by care managers so that the elderly individual can continue to live independently.

### Demographic variables of the elderly individuals

Demographic factors included age, gender, care-level, and cohabitation. The care-level is the level of care needed by a client as decided in the Long-term Care Insurance program. There are five levels of care need. Care managers were asked to fill out the Katz index of independence [24] and the Japanese Independence Index of Dementia on the clients [25]. Using the Katz index of independence, we assessed the elderly individual's difficulty in performing daily activities such as bathing, dressing, using the toilet, incontinence, mobility, and eating. The Japanese Independence Index of Dementia established by the Japanese Ministry of Health and Welfare was used to assess the severity of dementia. With the use of this index, care managers determined whether the elderly individuals needed help in their daily lives due to the symptoms of dementia. Thus, we were able to respond to various questions asked by the care managers about the need for utilization of the following home care services: home visiting nursing service, home help, and home rehabilitation.

### Medical procedures

According to previous studies, home visiting nursing service is not always required by clients managing medical procedures at home, and the necessity of home visiting nursing service differs according to the type of medical procedure [16]. Several medical procedures, such as items from accreditation criteria in long-term care need, were added to the questionnaire [26].

### Manageability of medical procedure or disease state by the client or family

The degree of manageability of a medical procedure or disease state by the client or family was a questionnaire item. The care manager chose from “Manageable,” “Manageable with assistance,” or “Unmanageable (including live-alone).”

### Severity of illness

The following items, “Terminal stage,” “Hospitalization within the past 6 months,” “Indication of dehydration within the past 6 months,” “Intermittent fever for the past 6 months,” and “Rapid severity of illness.”

### Caregiver variables

Regarding problems in caring, “Primary caregiver has health problems/is working/lives separately/is aged” and “Absence of secondary caregiver” were asked. For “Caring time” provided by the primary caregiver, the care manager chose from 4 categories: constantly, several hours per day, several days per week, and none. Caregiver factors included the primary caregiver's age, gender, family relationship, living situation, and caregiving experience, and the frequency with which care was provided by a secondary caregiver.

## Statistical analysis

### Identification of factors related to the need of home visiting nursing service

First, in order to select assessment items based on factors related to the need of home visiting nursing service, univariate analyses (chi-squared test, Fisher's exact test, unpaired *t*-test, Mann–Whitney *U*-test) were conducted for each item of the questionnaire.

Second, logistic regression analysis was subsequently carried out. A significance level of 0.05 was applied by univariate analysis to identify factors related to the need of home visiting nursing service (independent variable = items that were statistically significant in univariate analyses; dependent variable = need of home visiting nursing service). If two independent variables were highly correlated with each other, we selected one of them to avoid multicollinearity issues. However, as a result of univariate analyses, all care managers had judged “home visiting nursing service needed” when the client applied for assistance with one or more of the following five medical procedures: “Intravenous Hyperalimentation,” “Respirator,” “Tracheotomy care,” “Sputum aspiration,” and “Stoma management – unmanageable.” It was deemed that patients who received these five medical procedures, required home visiting nursing service, and the five items were excluded from logistic regression analysis. Data were analyzed using SPSS version 17.0 for Windows. The five items were added to the HVNS-NAF.

Finally, to create the HVNS-NAF, multiple logistic regression analysis was again conducted to determine the weight of each item. The final model contained all of the significant independent variables that had sustained significance (*p* < 0.1) in the first multiple logistic regression. In addition, we asked seven home visiting nurse managers by telephone and fax, whether the draft questionnaire lacked any items, and “Catheter care” was added according to their opinion. After rounding the regression coefficient to the nearest whole number, the smallest value was set as one point, and scores of the other items were determined according to their logarithmic value. The total score for each client was calculated as the sum of the weights of each item that applied to the client, and receiver operating characteristic (ROC) curves were used to determine the optimal cutoff value.

We then calculated the sensitivity and specificity for each cutoff value, and we defined the cutoff value as the score with the highest sensitivity and specificity. Based on the coefficients of these variables in the multivariate model, the score for each client was calculated as the sum of the coefficients for each item that applied to the client.

## Results

### Demographic characteristics of the clients

Table 1 summarizes the demographic characteristics of the clients. Among the 1085 the elderly clients requiring long-term care of care manager agencies in eight districts in Japan were studied, 380 (35.0%) needed home visiting nursing service. The average age of the 1085 clients was 81.6 years. Regarding gender, 62.2% were female. With regard to Activities of Daily Living (ADL) index, 79.0% of clients needed help in dressing, 45.3% needed help for mobility, and 48.2% needed help in eating. The most common disorders were cardiovascular disorders (34.8%), followed by musculoskeletal disorders (17.0%) and neurological disorders (14.5%).

### Selection of items for the HVNS-NAF

**Figure 1.**
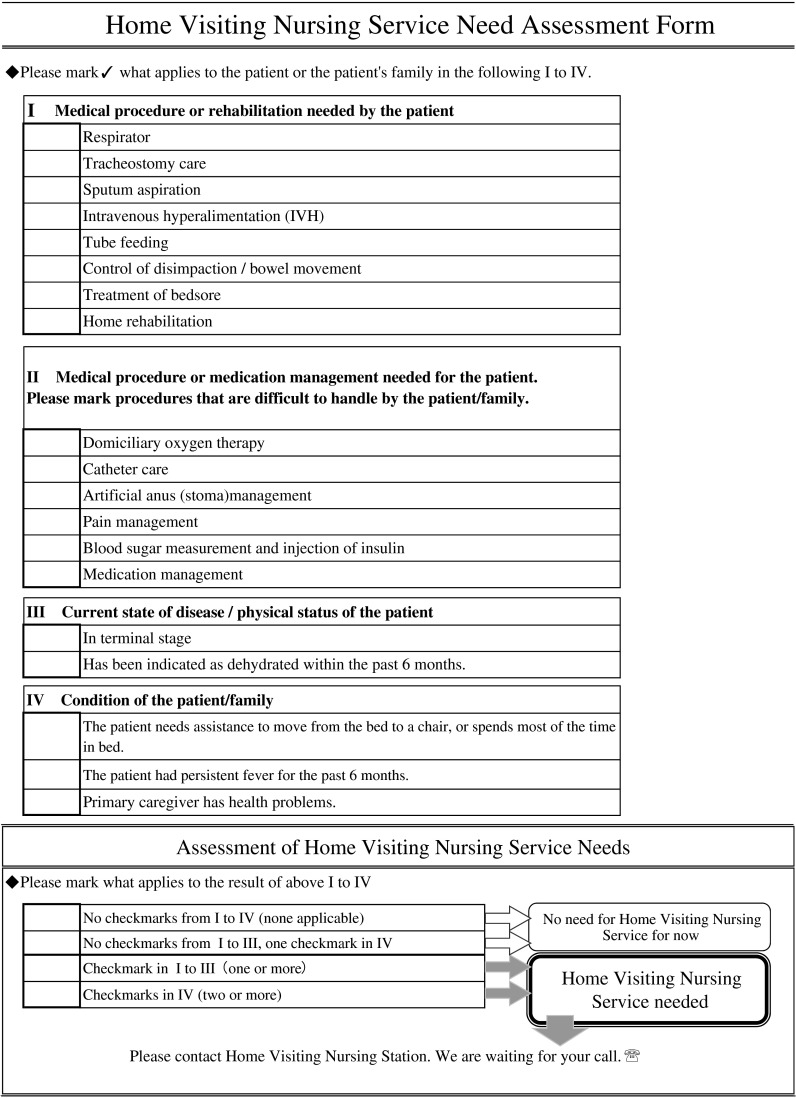
HVNS-NAF that was developed based on our analysis.

**Figure 2.**
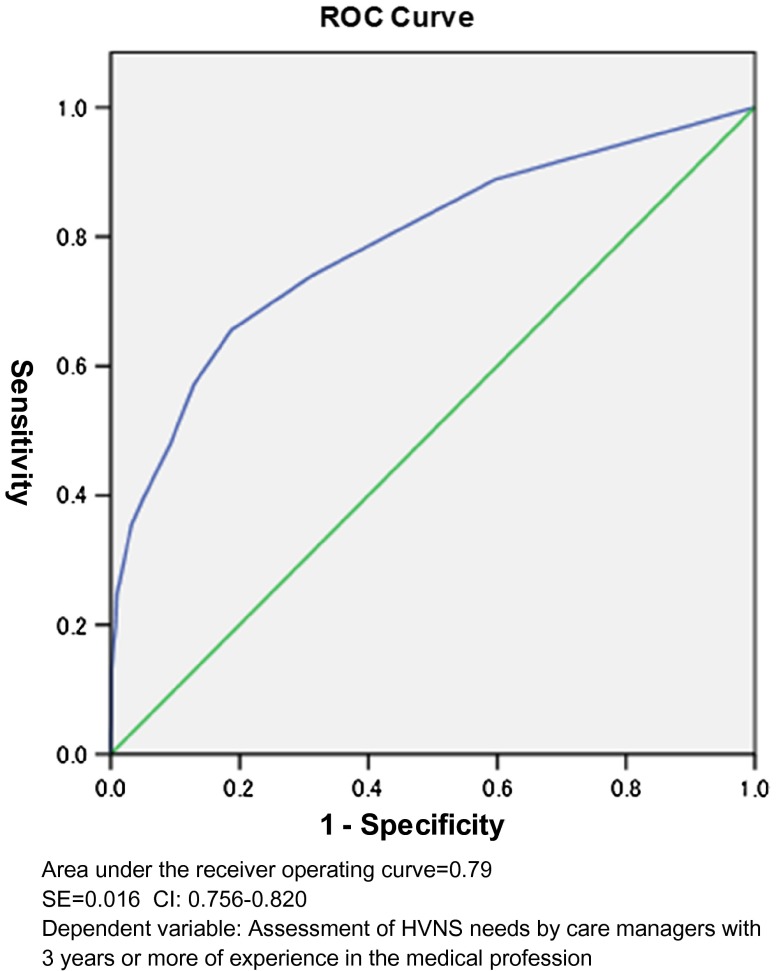
ROC curve.

In the end, 19 items were selected for the HVNS-NAF (Figure 1). Based on the coefficients of these variables in the multivariate model, the weight of each item was calculated (Table 2). The maximum score of the HVNS-NAF was 43 points. Next, we set the cutoff value, and plotted the ROC curve to consider its sensitivity and specificity (Figure 2). If the peak of the upper left shoulder of the ROC curve was the cutoff, we estimated that two or three points would prove feasible. The area under the ROC curve (AUC) was 0.79. When the cutoff value was two points and above, sensitivity was 77.0%, specificity was 68.5%, and positive predictive value (PPV) was 56.8%. When it was three points, sensitivity was 69.4%, specificity was 81.2%, and PPV was 66.6% (Table 3). In this study, we focused on identifying the potential need of home visiting nursing service and thus adopted the cutoff value of two points so as to prioritize high sensitivity. The PPV fluctuates according to the prevalence rate. The prevalence rate is the percentage of clients with mobility. In the clients with dependent mobility, PPV reached 63.5%. On the other hand, in the clients with independent mobility, PPV reached 47.5%.

## Discussion

The aims of this study were to clarify the characteristics of clients who need home visiting nursing service and to develop the HVNS-NAF. We developed the HVNS-NAF, which was composed of 19 screening items that characterized clients’ need for home visiting nursing service.

Of the 19 items, 14 screening criteria were related to medical procedures and home rehabilitation. The correlation between the incidence of home visiting nursing service need and particular medical procedures showed similar findings in previous studies [7, 16, 27]. Home visiting nursing service is not always needed when clients are scheduled for a medical procedure. Therefore, it is important to examine the relationship between each medical procedure and the need for home visiting nursing service.

Next, the difficulty in performing daily activities was related to home visiting nursing service need. Individuals with care-level 5 (lowest degree of independence) comprised the largest proportion among those receiving home visiting nursing service [28]. The care-level of users of home visiting nursing service was higher than the care-level of users of home care workers. It is important to evaluate how well the client can perform daily activities when assessing the need for home visiting nursing service.

Clients in the terminal stage of a disease needed home visiting nursing service. Previous studies reported that home visiting nurses were able to perform their roles as effectively for clients in the terminal stage [29, 30]. Home visiting nursing service can provide clients and families not only pain management and the most suitable care to clients with unstable illness, but also mental care. On the other hand, it was pointed out that clients did not receive the full benefit of home visiting nursing service [31]. Increasing numbers of users of home visiting nursing service in the terminal stage were expected by utilizing HVNS-NAF.

Furthermore, “symptom of dehydration” and “intermittent fever” within the past 6 months were related to home visiting nursing service need. Dehydration is a symptom of low-nutrient condition. Fever is a symptom of an infectious disease such as aspiration pneumonitis. These symptoms are especially important for assessing whether severe disease will develop. When having recurrent fever, it is crucial to determine the cause of the fever from its pattern, and to prevent the illness from becoming worse [31]. It was actually reported that home visiting nursing service prevented hospitalization for assessing fever and providing preventive care [26].

“Primary caregiver has health problem” was related to home visiting nursing service need. Excessive burden on the family caregiver causes health problems. Eventually, it will become difficult for the client to manage care at home. Because home visiting nurses provide assessment of not only the clients’ health problems but also the family's health problems [22–32], this item is important.

With regard to the HVNS-NAF, when the cutoff value was two points, with two points and above indicating that the client needs home visiting nursing service, the sensitivity was 77.0%, specificity was 68.5%, and PPV was 56.8%. These values of sensitivity and specificity seem to indicate that improvements need to be made. However, after considering that it has been difficult to standardize the need for home visiting nursing service in the past, these values of sensitivity and specificity seem to be valid. Because home visiting nursing service was related to a wide variety of variables, care managers did not have a systematic way of making a decision for the need of home visiting nursing service. From now on, verbalizing an equivocal concept of need for home visiting nursing service will be needed to clarify other variables related to home visiting nursing service. Since the sensitivity was low in the independent mobility group, it is also necessary to explore the associated factors specific for this group.

Several limitations of our study should be noted. First, it was difficult for care managers to make a decision about the need for home visiting nursing service without knowing the economic circumstances of the client and available services in the community. That is, these data were affected by the economic circumstance of the client and available services in the community. In addition, the small sample is a limitation of this study. The incidence of the need for home visiting nursing service was low. More studies with a larger sample size will be needed to better understand the complexities of utilization of home visiting nursing service by elderly clients and their families. Moreover, not only verification of validity but also verification of reliability is required from now on. While this study has several limitations, we could clarify characteristics of clients who need home visiting nursing service. Development of the HVNS-NAF is the first attempt at creating a standard based on the characteristics of clients who utilized home visiting nursing service. By using the HVNS-NAF, care managers who are novices and have little knowledge about home visiting nursing service, will possibly be able to make an appropriate decision on the need for home visiting nursing service. In countries where services for the elderly are provided by public funds as in Japan, it is beneficial to standardize the necessity of home visiting nursing care services. There are places in Japan where there is no care manager for elderly people. Also, since care managers are not always professional nurses, it is difficult for them to understand the meaning and importance of nursing care for their clients. Moreover, by introducing nursing care to elderly residents who wish to be hospitalized or re-hospitalized even though they can receive medical care at home, it becomes possible to find potential needs of home visiting nursing care and it is expected that these elderly residents can continue to live at home, thereby reducing medical expenditures. Furthermore, using the HVNS-NAF may reduce the unmet need for home visiting nursing service and prevent hospitalization.

## Conclusions

In this study, we developed a preliminary model that can be used to identify the need for home visiting nursing service. Development of the HVNS-NAF was the first attempt at creating a standard based on characteristics of clients who needed home visiting nursing service. This standardization of the decision about the need for home visiting service would lead to further improvements in integrated care for older people. Furthermore, using the HVNS-NAF may reduce the unmet need for home visiting nursing service and prevent hospitalization.

## Financial support

Financial support for this study was provided by the Health Care Science Institute in Japan. The authors report no conflicts of interest.

## Figures and Tables

**Table 1.**
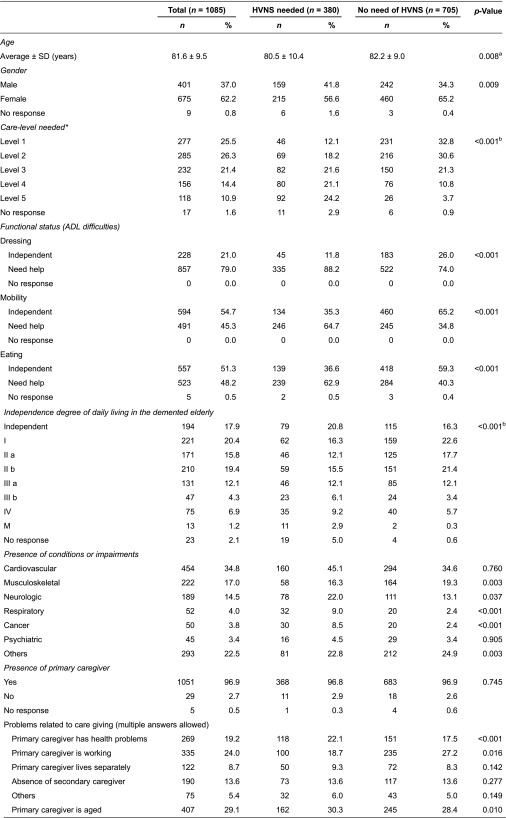
Characteristics of elderly clients requiring long-term care according to the need for home visiting nursing service

* Care level needed = A limit to the amount that can be expended for home long-term care.; “Level 1” = 165,800 yen per month, “Level 2” = 194,800 yen per month; “Level 3” = 267,500 yen per month; “Level 4” = 306,000 yen per month; “Level 5” = 358,300 yen per month.

^a^Unpaired *t*-test.

^b^Mann–Whitney *U*-test.

no mark: Chi-squared test.

**Table 2.**
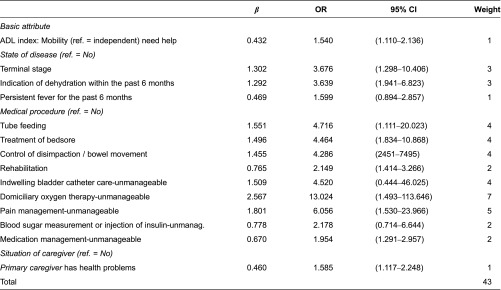
Results of logistic regression analysis and weight in HVNS-NAF

*β*, Regression Coefficient; OR, Odds Ratio; CI, Confidence Interval 95%.

The scores of items in the Home Visiting Nursing Service Need Assessment Form were decided according to the results of logistic regression analysis when each item of the Home Visiting Nursing Service Need Assessment Form was an independent variable, and home visiting nursing service need was a dependent variable.

**Table 3.**
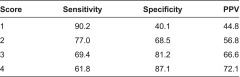
Sensitivity, specificity and PPV of HVNS-NAF (*n* = 1085)

Notes: Score of HVNS Assessment Form (range, 0–43); Dependent variable: Assessment of home visiting nursing service needs by a care manager with experience of 3 years or more.
